# On Poisoning with Arsenic

**Published:** 1818-01

**Authors:** J. Hume


*For the London Medical and Physical Journal.*

### *On Poisoning with Arsenic:* by J. HUME, Esq.

MORE than twenty-eight years have now elapsed since I first discovered the excellence of *silver* as a test for arsenic. This, like all other discoveries, has been, especially under my own guidance, gradually improving, through the aid of experiments founded on observation, and called for by numberless objections of the most absurd or frivolous nature, made by others; and some that really have an apparent foundation, unless the operator be on his guard, to distinguish a mere delusion from truth, supported by faithful and correct experiments.

It is with great reluctance I again return to this subject,—the defence of the tests and various methods of operating with them, which, from time to time, I have contrived and recommended in this and other periodical Journals. The great importance of the subject and the welfare of society form a sufficient apology; and, I trust, others will co-operate with me in canvassing, and with temperance, the questions now before the public,—Whether any test of arsenic is infallible?—Whether nothing short of the re-production of the poison in its *metallic* state can, or, in criminal cases, ought to be depended on?—Whether phosphates or phosphoric acid are always, or ever present in any of the animal fluids *in such a state* as to yield a *yellow* precipitate with silver?—Whether a decoction of onions can baffle all the powers of chemistry to detect arsenic?—I might add to this list another more extraordinary question, arising from the reflections upon a late trial, made by two of your correspondents, Mr. Kerr of Great Yarmouth, and Aminicus,*—Whether rabbit with onion-*sauce*, together with all the super-strata of salt, bread, wine, beer, cocoa, medicines, and other *et ceteras*, could so far resist, for a considerable period, the violent vomiting, retching, and other evacuations, as to retain their seat with so much obstinacy and effect as to be converted into onion-*decoction*?

I had almost been tempted to rest here, but that my silence would be considered as consent, and that no farther investigation would be undertaken by many scientific men who are so competent to decide, whether we are, in future, to be puzzled in our enquiries by decoction of onions, or even phosphate of soda; and whether thousands of men, women, and children, may be poisoned with impunity, when, in no single instance, the resuscitation of the metallic arsenic can be shown, so as to satisfy the minds of a jury, however complete the collateral and circumstantial evidence may be proved before a court of justice.

My time will not at present permit me to offer all the remarks upon the many palpable errors and unfounded opinions I have lately read, and which can very easily be set aside; the following remarks, although hastily drawn up, will serve, however, as a preface to a more extensive enquiry; they will show, that assertions are inconclusive and must ultimately misguide, and that clumsy and wilfully-erroneous experiments have been foisted into arguments to prove the fallibility of every test and method hitherto devised by others as well as myself, for proving the existence of arsenic.

I beg to ask, why one of the tests which I have recommended, the *ammoniaco*-nitrate of silver, was not taken into Mr. Kerr's or Dr. Neale's consideration, on following up, as is pretended, the experiments of Dr. Edwards? This test

* Medical and Physical Journal, Aug. p. 92-95.

renders a decoction of onions quite *red;* and, unlike the *simple* nitrate, it has no decided effect on phosphate of soda, it yields no *yellow* precipitate. Here, then, is one way of proving a most essential point, the *absence* of phosphate of soda. It is necessary that I should here say, the assumptions of Mr. Kerr, Dr. Neale, and some other gentlemen, that phosphates or phosphoric acid must always interfere with the operation, and that, consequently, in all juridical enquiries, the *yellow* or *green* precipitate (by silver or copper) can be no evidence, are void of support. Not one of the animal fluids, even those generated in and occupying the *primæ viæ*, contains a phosphate in *such a state* as to give at once a *yellow* precipitate with a proper *silver*-test. The great similarity which is said to exist between the *phosphate* of copper and the *arsenite* of this metal, in some cases, goes for nothing; it arises from an optical deception, that of viewing a *blue* precipitate through a *yellow* medium. If the precipitates be first *washed*, which is indispensably required, Mr. Kerr will have a more decided difference. But, if any one be at a loss to know one from the other, this gentleman has himself furnished us with a very ready process—exposure on red-hot iron. Not that this method meets with my approbation; it may do very well with a *lump* of the substance to be examined, but it is not the most eligible way to gain the *white fumes* from a small portion, perhaps scarcely visible.

Where the quantity of arsenic is extremely minute, or suspected to be so, it may even be advisable to *add* some of these very objectionable substances, the phosphates or muriates, to a solution, so that a more tangible shape may be given; or, in other words, the precipitate magnified and thus made more obvious to the senses. How to disentangle the arsenic itself from such a mixture as a phosphate, muriate, and arsenite of silver, is a task that I have ventured to perform with success; and, should I fail on any *interesting* occasion, let my evidence, as all other doubtful testimony should, go to the merciful scale of the balance.

If, in order to shew the fallibility of a test, some experiments are resorted to, I should expect these not to be on *one* side of the question only. Thus, barytes and lead may be confounded, by dropping into a solution of either of these bodies in any acid, a solution of sulphate of soda, and drawing this inference that, because, in both vessels there is in all appearance the same *white* and dense precipitate, the test is fallible. Just so might a false conclusion be drawn, were similar steps pursued with two solutions, phosphate of soda and arsenite of potash; a piece of dry nitrate of silver, being presented for a moment to the surface of either of the liquids, would certainly give, in resemblance, the *yellow* and plentiful precipitate; and a by-stander would consequently have his faith shaken respecting the validity of the claims in favour of the silver test.

Take an opposite position, in this way. Let two glass vessels be charged, one with phosphate of soda, the other a simple solution of oxide of arsenic, and, as all these experiments ought to be, both made with distilled water. Now, apply the dry nitrate of silver, as before, to the phosphate, and the same yellow precipitate will appear; but no such effect will happen to the solution of arsenic. A separate piece of the caustic should be taken in these experiments, to avoid error; for the same morsel that has been dipped into the phosphate should not be suffered to touch the arsenical solution. Any slight opacity in the *simple* solution of arsenic, on the contact with the caustic, is not to be regarded as arising from any union with arsenic; the *opaline* appearance, of which Mr. Kerr and Dr. Neale speak, is chiefly occasioned by some trifling impurity. Being now convinced that there is no yellow precipitate yet generated, let the operator hold a piece of blotting-paper, slightly moistened with a solution of ammonia, and nearly over the surface of the arsenical fluid, at the same time moving the vessel so as to cause an undulation, and there will instantly form a copious yellow indication of the presence of arsenic.

These experiments may be farther illustrated and substantiated by a great deal of very strong collateral evidence, and by more than one method: I shall now only mention one of these, and for that we are indebted to Dr. Ayrton Paris.

Either let two of these parallel experiments, which I need not repeat, be made upon two separate pieces of clean writing-paper; or, otherwise, spread a little of the freshly prepared arsenite of silver on one piece, and of phosphate of silver on the other. Suffer these to remain exposed till dry, and the phosphate of silver will gradually assume a *black* colour, or nearly so; while the other, the arsenite of silver, will pass from its original vivid yellow to an Indian yellow, or nearly a *fawn* colour. I have lately repeated these experiments to my entire satisfaction. Probably the summer season and more sun may influence the result, and create a trifling difference; I am, however, of opinion that this elegant proof forms a powerful auxiliary, and ought to be admitted as such. If these two experiments be performed with due skill and precision, another distinctive peculiarity will occur, which ought to be watched. Proceed in this way:—pour about a dram, or more, of the solution of phosphate of soda on one piece of writing-paper; and, to save time, as much of the arsenite of potash on another piece; then to each of them present a separate and small stick of dry nitrate of silver, drawing it backwards and forwards so as to disperse the colour. The first appearance to be remarked is, that the arsenite of silver puts on a yellow *floculent* figure, the colour curdling, accumulating, and assembling together in spots, as if it were a *yellow muriate* of silver; while the phosphate offers to our view a homogeneous colour, totally different in respect to texture, being quite smooth and uniform, as if it were laid on with a brush. There is also, at first, an evident difference in the colour: on this, however, I do not insist as a material point to the question at issue, for I have not yet sufficiently attended to the progressive change, which is more rapid than I had expected.

Your correspondent, Aminicus, boasts, that such is the perfection of the chemical art, that "even a *single grain* of the white oxyd of arsenic" will serve the purpose for a *metallic* exposure of the poison. Is this, then, the ultimate minimum to which we must be confined? Does this gentleman know how many infants may be murdered, I may say, with impunity, (granting him his position,) by a single grain of arsenic, or, what is equivalent, nearly three-hundred drops of Fowler's solution?* Would he venture to give the *whole* grain to a delicate female, or even to the most athletic prize-fighter?

After quoting what your correspondent, Mr. Crowfoot, is pleased to denominate "*the admirable test of Mr. Hume,*" Mr. Kerr asks, rather in a tone of exultation, "who will have confidence in this test in future?" I will answer only for myself,— that those persons who are open to conviction, understand the principles on which all my plans are founded, can operate without blunders or slovenliness, and are perfectly free from partiality and prejudice, are alone the judges of my pretensions.

To make any direct comment upon the scientific part of the evidence taken at that trial, would lead me to say more than is now necessary, or can be compatible

---

* The *solutio arsenicalis* of Dr. Fowler is not always prepared of sufficient strength to accord with prescription; this arises from the arsenic being adulterated with sulphate of barytes, gypsum, &c. It should not be purchased in the state of *powder.*

with the sentiments of Mr. Kerr, and of those whom he supports. A more luminous, impartial, and concentrated charge could not have been given to any jury, than that which was delivered by the learned judge (Mr. Justice Abbott).—"You will observe," said this excellent judge, "that his (Dr. Neale's) experiments were made with onions in a very *different* state from what rabbits boiled with onions usually are.—They merely contradict the opinion of Dr. Edwards, without seeing or knowing the appearances which he saw and knew.—It is clear that *he* (Dr. Edwards) came in too late to do any good upon this occasion, &c."

I have here given a short sketch of what I would oppose, particularly to Mr. Kerr's remarks, and to that species of simultaneous opinions of some practitioners, who seem determined at all hazards not to be convinced; otherwise the expressions, "I am borne out by all philosophical chemists in this country,—I am borne out by the best authorities in the country," &c. "that the only test that can bear a man out in swearing to the presence of arsenic, is the reproduction of the metal in its metallic state,"—with some other general, yet vague and unfounded, opinions, which never met with the universal assent of men of science of the present age, especially in this country.

Before I conclude, it will be proper to refer your readers to some of the principal parts of my former communications to the public upon this very momentous question: I shall, therefore, now proceed, by briefly enumerating the dates of such of my letters as, I think, Mr. Kerr and others ought to consult before they attempt to lull the whole community into so fatal a security, as to believe that nothing short of a revivification of the arsenic into its *metallic* form ought to be accepted as evidence in cases of medical jurisprudence. Of the existence of the greater part of my papers, Mr. Kerr, Dr. Neale, and many others, seem altogether ignorant: to them, therefore, I recommend a careful perusal of those I shall now mention.

The first public communication, dated May 19th, 1809,* announced *silver* to be the most effectual test for detecting arsenic. It was a short letter, not occupying more than one page of the work in which it appeared. As it is evident that the operation must be by *double* chemical affinity, or elective attraction, I then advised subcarbonate of *soda* to be joined to the arsenic, and nitric *acid* to the silver; dry nitrate of silver (lunar caustic) being always at hand in every medical

* Philosophical Magazine.

shop, renders this salt at all times the most convenient and eligible preparation. My next paper,* May 14th, 1810, will be found in your own valuable Journal; where I ask one of your correspondents, Mr. Jones, "was there any sub-carbonate of *potash*, or any other *alkali*, employed, previous to the admixture of the sulphate of copper?" As in this question the word "alkali" was in italics, I trust that *ammonia* could not be denied its place; and hence it is quite clear, that no alkali could afterwards be chosen for the purpose, and deemed a *discovery*;—the hint is too broad to be mistaken. Soon after the last paper, I wrote another (July 13, 1810,)† for this Journal, giving a new method for practice,—that of converting the arsenic, or its compounds, into an *arseniate*, either as a preliminary process, or inductive to ulterior means; and this to be accomplished by the aid of *nitrate of potash*. *Magnesia*, *barytes*, *lime*, and other earths, have been proved to be eligible tests for arsenic. My experiments on them will be found in my communication, dated October 14th, 1812.‡ Nearly two years after I had pointed out that alkalies in general might be employed, I was induced to make various experiments with *ammonia*,§ particularly as a very celebrated and intelligent chemist, Dr. Marcet, seemed to give it the preference. These trials led me at once to form a *triple* salt, with either silver or copper and this alkali, which I now consider as a most valuable acquisition to analytical chemistry; for, by this assistance, we are able to ascertain the precise quantum of alkali required, which could not otherwise be readily obtained. This test possesses another property, which I have already stated above,—it does not affect phosphate of *soda*: other phosphates, I know, exist in the human system, especially in the urine; and, as the bones are almost entirely a phosphate of lime, which is quite insoluble, I believe we have very little to fear from the presence of any phosphate whatever in fluids, mixed with extraneous matters, ejected from the human stomach.

*Long Acre; Dec.* 12, 1817.

---

* London Med. and Phys. Journ. † ib. ‡ Philos. Mag.

§ For this communication, see Med. and Phys. Journal, and Philos. Magazine. My letters are Aug. 9th and 10th, 1812.

